# Poison frogs rely on experience to find the way home in the rainforest

**DOI:** 10.1098/rsbl.2014.0642

**Published:** 2014-11

**Authors:** Andrius Pašukonis, Ian Warrington, Max Ringler, Walter Hödl

**Affiliations:** 1 Department of Cognitive Biology, University of Vienna, Althanstrasse 14, 1090 Vienna, Austria; 2 Department of Integrative Zoology, University of Vienna, Althanstrasse 14, 1090 Vienna, Austria

**Keywords:** amphibian navigation, amphibian learning, spatial cognition, Dendrobatidae, spatial orientation

## Abstract

Among vertebrates, comparable spatial learning abilities have been found in birds, mammals, turtles and fishes, but virtually nothing is known about such abilities in amphibians. Overall, amphibians are the most sedentary vertebrates, but poison frogs (Dendrobatidae) routinely shuttle tadpoles from terrestrial territories to dispersed aquatic deposition sites. We hypothesize that dendrobatid frogs rely on learning for flexible navigation. We tested the role of experience with the local cues for poison frog way-finding by (i) experimentally displacing territorial males of *Allobates femoralis* over several hundred metres, (ii) using a harmonic direction finder with miniature transponders to track these small frogs, and (iii) using a natural river barrier to separate the translocated frogs from any familiar landmarks. We found that homeward orientation was disrupted by the translocation to the unfamiliar area but frogs translocated over similar distances in their local area showed significant homeward orientation and returned to their territories via a direct path. We suggest that poison frogs rely on spatial learning for way-finding in their local area.

## 1. Introduction

Repeated attempts have been made to formulate a conceptual and evolutionary framework for animal navigation, integrating the findings from birds, mammals and insects [1,2]. Among vertebrates, comparable spatial learning abilities have been found in birds and mammals, as well as some turtles and teleost fish. Furthermore, potentially homologous pallial regions responsible for spatial learning have been identified in all of these taxa [3]. Meanwhile, very little is known about spatial learning and navigation in amphibians—the root branch of all tetrapods.

Spatial memory and its flexibility are expected to vary with a species' mode of locomotion, home-range size, habitat complexity and the variability of the resources being exploited. Overall, amphibians are the most sedentary vertebrates (for a review, see [4,5]), but some of them face challenging navigational tasks. Poison frogs (Dendrobatidae) are restricted to the highly structured Neotropical habitats where they show some of the most complex spatial behaviours among amphibians. These small territorial frogs with terrestrial clutches rely on water for tadpole development. Consequently, poison frogs routinely shuttle tadpoles on their back from the terrestrial clutches to suitable deposition sites, many of which are temporary and widely dispersed [6].

Homing after experimental translocations has been observed in many amphibians (for a review, see [7]), but to date the research on amphibian orientation has largely focused on the sensory basis (e.g. [8,9]) and not the cognitive mechanisms. Some amphibians use large-scale directional cues, but such cues cannot account for accurate way-finding within a few hundred metres [10,11]. Several authors have suggested that learned local cues might be important for amphibian homing performance (e.g. [7,12]), but empirical evidence is lacking. We hypothesize that dendrobatid frogs rely on experience with the local area for flexible way-finding in their complex habitat.

There are three main challenges to testing the dependence on experience for navigation in the field: (i) finding an animal strongly motived to orient towards a goal, (ii) tracking the movements, and (iii) being able to quantify or exclude the familiarity with a given area. We overcame them by (i) experimentally displacing highly territorial males of a poison frog, *Allobates femoralis*, (ii) using a harmonic direction finder with miniature transponders to track these small frogs in their natural habitat [13], and (iii) using a natural river barrier to separate the translocated frogs from any learned local cues.

## 2. Material and methods

*Allobates femoralis* is a small, territorial, leaf-litter frog common throughout the Amazon basin and the Guiana Shield [14]. Translocated males return to their territories from up to 400 m [15]. To test whether experience with the local cues affects *A. femoralis* way-finding, we fitted 46 territorial males with miniature transponders and translocated them between 187 and 365 m away from their territories. To exclude the possibility of en-route path integration via visual, olfactory or magnetic cues, we transported frogs along an indirect path in an opaque, airtight container with a freely rotating rod magnet. Twenty-five frogs were released in the area occupied by the same population (i.e. mainland; mean translocation distance = 254 m, s.d. = 58.8 m), whereas 21 frogs were released at equivalent distances in the centre of an approximately 5 ha river island (i.e. island; mean translocation distance = 301 m, s.d. = 51.2 m) with an isolated population of *A. femoralis* (electronic supplementary material, figure S1). Both release areas provided comparable and suitable habitats for *A. femoralis*, but the frogs do not naturally cross the river barrier. Frogs released on the island had an area with a radius of about 100 m to move in before reaching the waterfront (see the electronic supplementary materials and methods for more details).

After the release, we tracked the individual movement patterns for about 7 days or until the frog returned to the home territory. We localized each frog every 15–60 min during their daylight activity period using a commercially available harmonic direction finder (RECCO R8, Recco AB). The position of each frog was recorded on a pocket computer (MobileMapper 10, Spectra-Precision) in ArcPAD (ESRI) using a detailed background map (see the electronic supplementary materials for the full tracking procedure). Experiments were carried out between 1 February 2014 and 22 April 2014 near the field camp ‘Saut Pararé’ in the nature reserve ‘Les Nouragues’, French Guiana.

For each frog, we analysed the X- and Y-coordinates of each position in ArcGIS 10 (ESRI), after averaging points that were spaced less than 1 m apart (estimated mapping error in the field). We normalized all movement vectors towards the home territory of each frog, so that 0° was the common home bearing. We further described trajectories by a straightness coefficient (SC), defined as the ratio of straight path length to total path length. To test for significance of homeward orientation, we considered only those frogs that moved at least 25 m away from their release point (*n*_island_ = 12, *n*_mainland_ = 14) and used a vector length weighted, second-order Hotelling's test (Oriana 4, Kovach Computing Services). The vector length corresponds to the SC until the point where the bearing was measured. The island and mainland bearing distributions were compared with Hotelling's two-sample test. For visual representation, we plotted home direction normalized trajectories derived from the linear interpolations of consecutive positions (see the electronic supplementary materials and methods for more details).

## 3. Results

**Figure 1.**
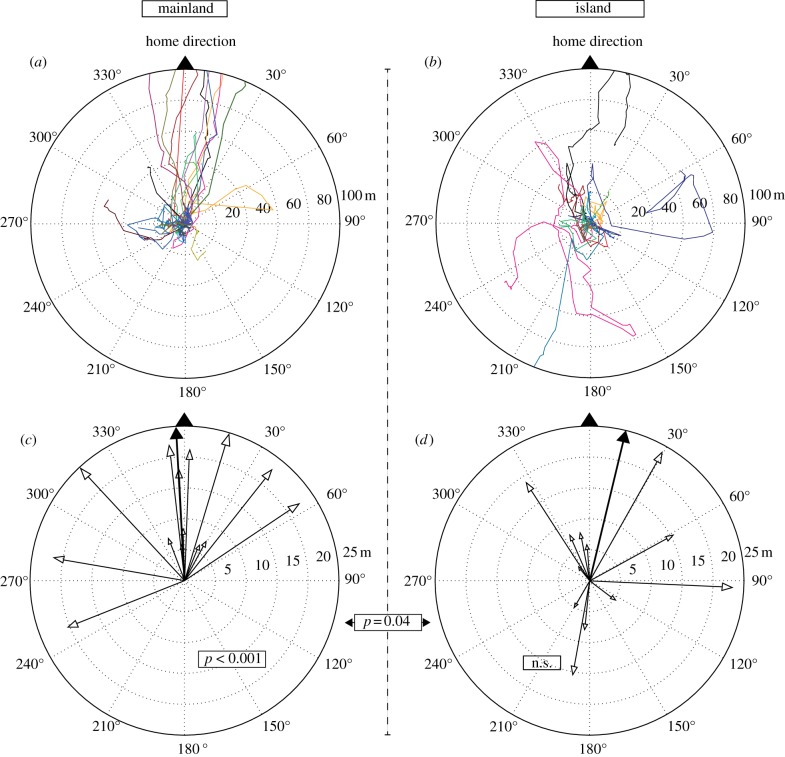
Polar plots comparing homeward orientation of frogs translocated to a familiar area, i.e. (*a*,*c*) mainland and an unfamiliar area, i.e. (*b*,*d*) island. (*a*,*b*) Full trajectories derived from linear interpolations of consecutive positions within 100 m from the release point on the mainland and the island, respectively. Each line represents a different individual. (*c*,*d*) Vector plots for individuals that moved at least 25 m from the release point (*n*_island_ = 12, *n*_mainland_ = 14). Vector direction corresponds to the home direction normalized bearing at approximately 25 m from the release point. Vector length corresponds to the path straightness until that point. Each white arrow represents a different individual, while the filled arrows show the mean orientation. Significance levels from the second-order Hostelling's test and a two-sample Hostelling's test are shown.

**Figure 2.**
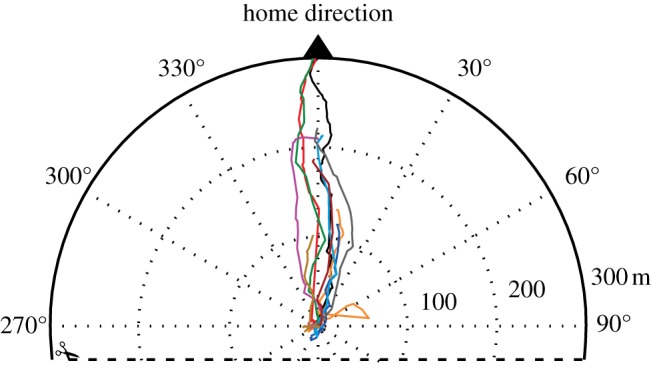
Part of a polar plot showing homing trajectories over 300 m of frogs translocated into the familiar area, i.e. mainland. All trajectories are home direction normalized. Each line corresponds to a different individual. Only individuals that moved at least 100 m away from the release point are shown (*n* = 10).

While on the mainland, frogs that moved at least 25 m away from the release point showed significant homeward orientation (*n* = 14, mean vector direction = 354°, 95% CI = 303.5°–30°, Hotelling's *F* = 14.6, *p* < 0.001; figure 1*a,c*), the frogs on the island showed no significantly preferred direction (*n* = 12, Hotelling's *p* > 0.1; figure 1*b,d*). At 25 m, there was a significant overall difference in bearing distribution of frogs on the mainland from those on the island (Hotelling's two sample *F* = 3.78, *p* = 0.038). On the mainland, seven frogs returned to within a few metres of their capture location and another three moved at least 100 m homewards. All 10 frogs that moved at least 100 m away from the mainland release sites were strongly homeward oriented (*n* = 10, mean vector direction = 7°, 95% CI = 356°–17°, Hotelling's *F* = 62.8, *p* < 0.001) and showed direct homing trajectories (Mean_SC_ = 0.77, s.d. = 0.18) (figure 2).

## 4. Discussion

The fact that frogs choose a direct path home when released in their local area but are disoriented in an unfamiliar area demonstrates that experience with local cues is necessary for successful homing. Our findings cannot be solely explained by the use of large-scale gradients or goal-associated cue guidance (e.g. distant visual, olfactory or acoustic beacons). They suggest a role of spatial learning.

True spatial learning requires processing and storing information about distances and directions. It allows novel routes to be created in the familiar area [2]. There are several ways to use learned cues that do not require spatial information processing. The most common non-spatial solutions include using goal-associated cues as beacons, using a sequence of intermediate beacons, or using cues as triggers for associated motor routines that bring the animal to the goal. All of these mechanisms allow an animal to orient along specific learned routes or when in direct sensory contact with the goal. Strong initial orientation and straight homing trajectories of homebound frogs are inconsistent with route-based orientation. We cannot claim with certainty that the performed homing paths were completely novel. However, we find it very unlikely that all homebound individuals had previously taken these short-cuts to their territories from the arbitrarily chosen release sites. Even though some males maintain stable territories for up to several months, the overall territorial system is dynamic and regular re-shuffling of positions occurs [16,17]. Inflexible, route-based navigation would require a rapid learning of multiple new routes over a large area whenever a territorial shift occurs. The type of homing observed in translocated *A. femoralis* is suggestive of a learned spatial map, but more experiments will be needed to test this hypothesis.

*Allobates femoralis* relies on the exploitation of multiple, widely dispersed, temporary aquatic deposition sites for successful reproduction. In a recent study, *A. femoralis* males were found transporting tadpoles up to 180 m away from their territory. In addition, males transported more tadpoles at once when going to more distant deposition sites, which suggests some spatial knowledge of the area [18]. Tadpole transport takes several hours or even days. This comes at a direct cost in the form of lost reproductive opportunities and a risk of losing the territory altogether. Under such circumstances, we expect a direct selection for a strong spatial memory and the ability to use it flexibly to find the optimal route to deposition sites and back home.

Qualitatively comparable spatial learning abilities have been shown in a broad range of vertebrates [1,3]. Amphibians are a key taxon in understanding vertebrate evolution but little is known about amphibian spatial cognition and the neural mechanisms behind it. Our findings suggest that among amphibians at least dendrobatid frogs rely on learning for successful orientation. Amphibians display an extreme diversity of reproductive behaviours [19] resulting in an extremely diverse spatial ecology. Thus, understanding the cognitive mechanisms behind amphibian orientation could give key insights into the evolution of spatial cognition.

## Supplementary Material

Supplementary materials and methods

## Supplementary Material

Figure S1

## Supplementary Material

Mainland data

## Supplementary Material

Island data

## Data accessibility

All positional data used for analysis have been uploaded as the electronic supplementary material.
