# Supplementary material for: Poison frogs rely on experience to find the way home in the rainforest
Source: Biol Lett. 2014 Nov;10(11):20140642. doi: 10.1098/rsbl.2014.0642 (PMC4261859; doi:10.1098/rsbl.2014.0642)
Supplement: Supplementary materials and methods [file rsbl20140642supp1.docx]

Supplementary materials and methods

Telemetry equipment

We used the harmonic direction-finding (HDF hereafter) telemetry technique to obtain homing trajectories of experimentally translocated, territorial *Allobates femoralis* males. The HDF system consists of a portable transceiver and a passive reflector (i.e. transponder). The transceiver emits a microwave signal and recaptures a harmonic frequency reflected from the tag attached to an animal, thereby providing directional information (for more details see [1,2]). The small size of the reflector tags allows this technique to be used on smaller animals than would be possible with conventional radio tracking (i.e. VHF telemetry). We used a commercially available transceiver (RECCO^®^ R8, Recco AB). The transponder consisted of a Schottky diode soldered between two antennas. Antennas were made of 40 μm steel strands forming a 3 by 12 cm T-shaped dipole with the braze point sealed in heat-shrink tubing and silicone. We attached the transponder to the frog using a waistband made of 1 mm diameter silicon tubing. The short part of the T-shaped antenna was secured inside the tube and the waistband was fixed with a cotton thread going through the tubing. The tag together with the attachment amounted to less than 5% of the frogs’ body weight (frog weight ~ 2 g, tag weight < 0.1 g).

Experimental areas

Frogs were sampled near the field camp ‘Saut Pararé’ (4°02’ N, 52°41’ W, WGS84) in the nature reserve ‘Les Nouragues’, French Guiana. The study area consists mainly of primary lowland rainforest bordering the ‘Arataye’ river to the south. The two experimental areas were the riverside surrounding the field camp (i.e., mainland) and a river island of approximately 5 ha in front of the field camp (i.e., island) (Fig. S1). The mainland sampling area sustains a population of at least 300 calling males distributed in patches. The area can be characterized as ‘terra firme’ forest with a complex terrain of small hills and ridges (~ 30 – 50 m a. s. l.) partitioned by swamps and creeks in lower areas. The island is mostly covered in ‘terra firme’ forest with less complex terrain (~ 30 – 40 m a. s. l.) and lacks any persisting creeks. It was previously uninhabited by any dendrobatid frog species, including *A. femoralis*. However, an experimental population of *A. femoralis* had been introduced in 2012 and has successfully established on the island. At the time of the study, the population had about 60 calling males, approaching the density of the mainland sampling area. The Arataye river forms a strict physical barrier between the two populations, as width and current strength do not allow for any intentional crossings of *A. femoralis* individuals. For this study, all frogs were sampled from the mainland population.

Choice of individuals and translocation procedure

Calling males were detected and identified as territorial if they showed stereotypical territorial defence behaviour (calling and phonotactic approach), which was elicited by broadcasting conspecific advertisement calls, simulating an intruder

[3,4]. Individuals could be recognized and identified by their unique ventral coloration pattern [5]. Frogs were captured with a transparent airtight plastic bag, weighed and photographed ventrally for identification. Their precise capture locations were recorded with the mobile GIS software ArcPad^TM^ 10.0 (ESRI) on a pocket computer (MobileMapper^TM^ 10, SpectraPrecision) using a detailed background map, based on a network of reference points and natural structures [6].

Each territorial male was equipped with a transponder and immediately released at its initial capture location. We revisited the territories of the tagged males over the next 36 h to confirm their territorial behaviour after tag attachment. Males were only used for translocation if they were observed calling, reacting aggressively to a simulated intruder, or in courtship with a female during at least one of the visits. Over the whole study period, we tagged 72 territorial males, of which 46 were used for the translocations.

Selected males were collected from their territories in the morning hours, translocated and subsequently released in the afternoon. Each frog was placed in an individual, clean, airtight plastic bag. All bags were placed in an opaque waterproof barrel equipped with a suspended, freely rotating neodymium rod magnet (Ø = 15 mm, length = 100 mm, residual magnetism = 1.17-1.21 T). The barrel was rotated multiple times after each frog was added, during transport and before the release. Between one and five frogs were transported at the same time. All frogs were transported to the field camp (Fig. S1) before being released at their corresponding release sites on the island or the mainland. The average time between capture and release was 210 min (SD = 33 min) for the mainland translocations and 194 min (SD = 62 min) for the island. The average translocation distance was 254 m (min = 187 m, max = 365 m) on the mainland and 301 m (min = 193 m, max = 364 m) on the island. Frogs released on the mainland were translocated away from the river (northeast, north or northwest) or towards the river (southeast, south or southwest), depending on the location of their territory (Fig. S1). Individuals were not translocated towards east and west to avoid their release across a water barrier such as a creek or a swamp. To achieve comparable translocation distances for frogs released on the island, we selected frogs with territories close to the river, resulting in translocations towards southeast, south, or southwest. Frogs translocated to the island were released approximately in the centre of the island, spaced out by at least 10 m when multiple frogs were translocated at once. All frogs were released at the base of understory palms (*Astrocaryum sp.*), which are often used as calling perches by territorial males.

Tracking procedure

Following the release, all frogs were continuously tracked during their daylight activity hours (~ 7 h 30 to 18 h 30). *Allobates femoralis* is exclusively diurnal and we never observed a frog move between 19 h 00 and 7 h 00. During the tracking hours, each frog was relocated every 15–60 min. Longer intervals occasionally occurred due to bad weather conditions and/or additional time needed to relocate a frog.

To locate the frogs, we followed the increasing amplitude of the reflected signal until visually spotting an individual. In cases of poor visibility or if an individual was hiding, we homed in to the signal within less than one meter. If an individual remained stationary and hidden for longer periods, we carefully uncovered the frog at least once a day to make sure that the tag had not fallen off and that the individual showed no injuries. Because the harmonic signal does not carry an individual signature, we colour coded the transponders. A frog’s identity was always visually confirmed if there was any ambiguity in the spatial separation of simultaneously tracked frogs. Occasionally, we handled the frogs to fix or replace a broken or miss fitting waistband or transponder. These manipulations never took more than a few minutes.

Every position fix was recorded on a background GIS map as mentioned above. Occasionally, frogs moved out of the mapped area, in which case we mapped the positions by measuring the distance and compass direction from the nearest mapped reference point. Distance was measured with a laser rangefinder (DLE 50; Bosch) and compass direction was measured with a precision compass (Suunto Tandem 360PC/360R DG, magnetic inclination zone 2; Suunto).

Initially, we planned to track all frogs for seven full days or until their return to the home territory. However, several factors determined shorter or longer tracking periods. On the island, shorter periods were forced by predation events (2 frogs after 3 and 6 days); injury (1 frog after 2 days) and lost transponders (1 frog after 3 days). On the mainland, 7 frogs returned to their territories in 3 to 5 days. In addition, the tracking period on the mainland was extended (8 to 14 days) for 8 frogs because of an extended dry period, which resulted in a very low mobility of frogs. Frogs that moved homewards after the first heavy rain were tracked until they returned to their home territory. All frogs were returned to their territories at the end of the tracking period and many of them were subsequently observed showing territorial behaviour.

Data analysis

Initial visualization, extraction of coordinates, and distance measurements were done in the GIS software ArcGIS^TM^ 10 (ESRI). The geographic coordinates of all locations were projected (UTM, zone 22N, WGS84) and extracted as X- and Y-coordinates in metres. We averaged consecutive coordinates of an individual when they were within less than one metre, which we considered to be our measurement error. We normalized all trajectories by shift and rotation to a relative zero release point and a single home bearing at 0°. We calculated a straightness coefficient (SC) as the ratio between the straight-line distance and the actual path distance, with a ratio of one indicating a perfectly straight path.

To test for initial homeward orientation, we considered only those frogs that moved at least 25 m away from their release point. We measured the bearing between the release point and the position closest to the 25 m-radius circle crossing. The SC was measured between the same two points. We used second-order Hotelling’s circular statistics test for a significant unimodal distribution of each sample, i.e. island and mainland. The test weights each angular measure by the vector length, which corresponds to the SC value in our data set (Oriana 4.02, Kovach Computing Services, Pentraeth, Wales, UK). In addition, we used the same procedure to test the homeward orientation of the mainland frogs that moved at least 100 m from the release point. The SC for the total homing path of these frogs was also calculated.

Ethical statement

Our study was approved by the scientific committee of the research station where the fieldwork was conducted. All necessary permissions were provided by the ‘Centre National de la Recherche Scientifique’ (CNRS) and by the ‘Direction Régionale de l’Environment de Guyane’ (DIREN). Permit numbers: ARRETE n°2011-44/DEAL/SMNBSP/BSP du 19/07/2011 and ARRETE n°2013-04/DEAL du 14/01/2013. All sampling was conducted in strict accordance with current French and EU law and followed the ASAB guidelines for the treatment of animals in behavioural research and teaching.

References

1. Gourret, A., Alford, R. & Schwarzkopf, L. 2011 Very small, light dipole harmonic tags for tracking small animals. *Herpetol R* **42**, 522–525.

2. Mascanzoni, D. & Wallin, H. 1986 The harmonic radar: a new method of tracing insects in the field. *Ecol Entomol* **11**, 387–390.

3. Hödl, W. 1987 *Dendrobates femoralis* (Dendrobatidae): a handy fellow for frog bioacoustics. In Proceedings of the 4th Ordinary General Meeting of the Societas Europaea Herpetologica: 17-21 August 1987; Nijmegen (eds J. J. van Gelder, H. Strijbosch & P. J. M. Bergers), pp. 201-204.

4. Ursprung, E., Ringler, M. & Hödl, W. 2009 Phonotactic approach pattern in the neotropical frog *Allobates femoralis*: A spatial and temporal analysis. *Behaviour* **146**, 153–170. (doi:10.1163/156853909X410711)

5. Ursprung, E., Ringler, M., Jehle, R. & Hodl, W. 2011 Toe regeneration in the neotropical frog *Allobates femoralis*. *Herpetol J* **21**, 83–86.

6. Ringler, M., Mangione, R., Pašukonis, A., Rainer, G., Gyimesi, K., Felling-Wagner, J., Kronaus, H., Réjou-Méchain, M., Chave, J., Reiter, K. et al. High-resolution forest mapping for behavioural studies in the Nature Reserve 'Les Nouragues', French Guiana. *J Maps* in press. (doi: 10.1080/17445647.2014.972995)

Figure S1. Map of the study area showing home territories of translocated frogs (coloured circles) and release sites (arrowheads) connect with lines. Contour lines (1 m) are in light grey; creeks and Arataye river in dark grey; swamps marked as tussock on the white background; field camp marked with a camping symbol. All frogs were transported to the field camp before being released at their corresponding release sites on the island or the mainland.
