# Supplementary figures and images for: Poison frogs rely on experience to find the way home in the rainforest
Source: Biol Lett. 2014 Nov;10(11):20140642. doi: 10.1098/rsbl.2014.0642 (PMC4261859; doi:10.1098/rsbl.2014.0642)

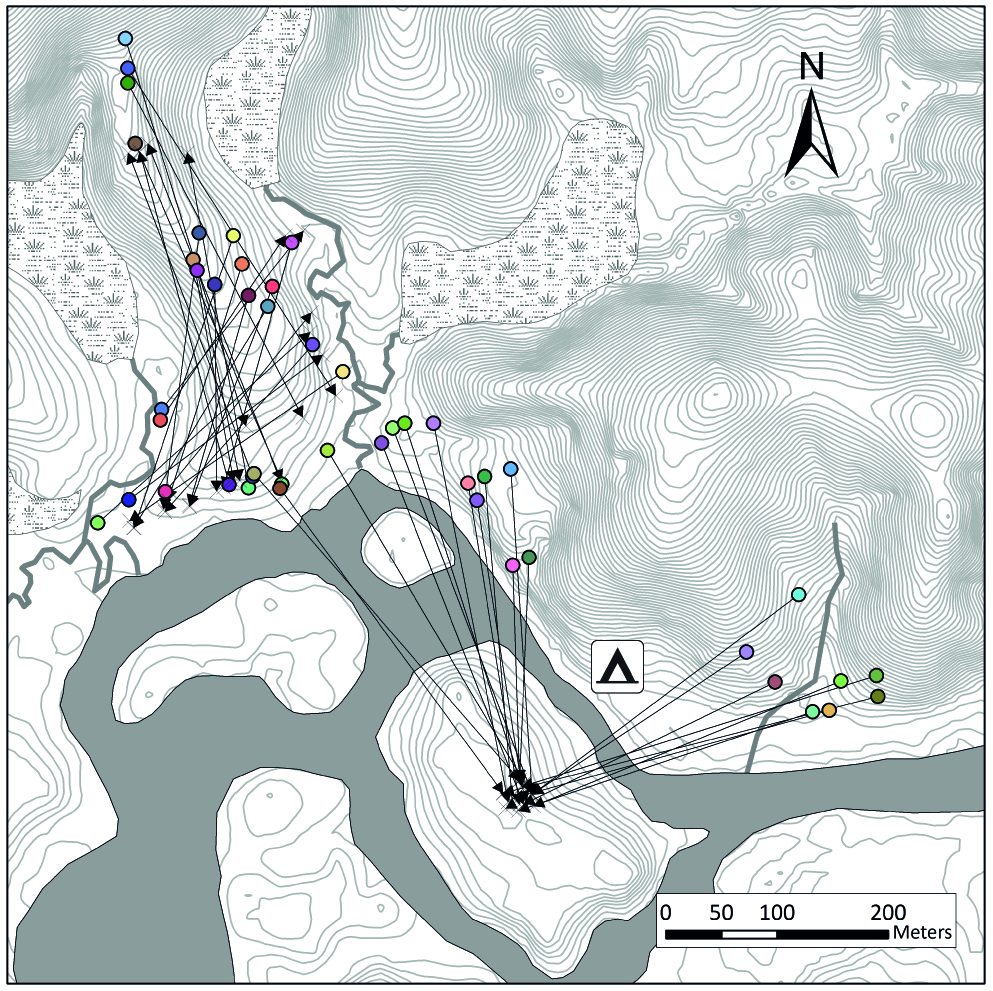

Supplement: Figure S1 [file rsbl20140642supp2.tif]
